# Prognostic value of members of NFAT family for pan-cancer and a prediction model based on NFAT2 in bladder cancer

**DOI:** 10.18632/aging.202982

**Published:** 2021-05-07

**Authors:** Zhou-Tong Dai, Yuan Xiang, Yundan Wang, Le-Yuan Bao, Jun Wang, Jia-Peng Li, Hui-Min Zhang, Zhongxin Lu, Sreenivasan Ponnambalam, Xing-Hua Liao

**Affiliations:** 1 Institute of Biology and Medicine, College of Life and Health Sciences, Wuhan University of Science and Technology, Wuhan 430081, Hubei, P.R. China; 2 Department of Medical Laboratory, Central Hospital of Wuhan, Tongji Medical College, Huazhong University of Science and Technology, Wuhan 430014, Hubei, P.R. China; 3 School of Molecular and Cellular Biology, University of Leeds, Leeds LS2 9JT, United Kingdom

**Keywords:** bladder cancer, prognostic risk score, overall survival, nomogram, NFAT

## Abstract

Bladder cancer (BLCA) is one of the common malignant tumors of the urinary system. The poor prognosis of BLCA patients is due to the lack of early diagnosis and disease recurrence after treatment. Increasing evidence suggests that gene products of the nuclear factor of activated T-cells (NFAT) family are involved in BLCA progression and subsequent interaction(s) with immune surveillance. In this study, we carried out a pan-cancer analysis of the NFAT family and found that NFAT2 is an independent prognostic factor for BLCA. We then screened for differentially expressed genes (DEGs) and further analyzed such candidate gene loci using gene ontology enrichment to curate the KEGG database. We then used Lasso and multivariate Cox regression to identify 4 gene loci (FER1L4, RNF128, EPHB6, and FN1) which were screened together with NFAT2 to construct a prognostic model based on using Kaplan-Meier analysis to predict the overall survival of BLCA patients. Moreover, the accuracy of our proposed model is supported by deposited datasets in the Gene Expression Omnibus (GEO) database. Finally, a nomogram of this prognosis model for BLCA was established which could help to provide better disease management and treatment.

## INTRODUCTION

Bladder cancer (BLCA) is one of the common malignant tumors in the human population, and has a frequency ranking of 10^th^ amongst the catalog of all malignant tumors worldwide [1]. BLCA is also the second most common malignant tumor found associated with the urinary system [2]. New BLCA cases worldwide accounts for 3% of total cancers with mortality accounting for 2.1% of total cancer-related deaths [1]. 2018 American Cancer Society statistics revealed over 1.7 million new cancer cases, with over 80 000 BLCA cases thus showing a high incidence of this disease state [3]. Notably, the pathophysiological properties of BLCA disease are exemplified by significantly increased metastasis, linked to the higher mortality rate. Conventional BLCA disease therapy combines both chemotherapy adjuvant and surgical resection of the tumor. In spite of such radical and invasive therapies, BLCA patient median survival time is ~15 months, with a relatively low 5 year survival rate of ~15% [4, 5]. There is thus an urgent need to identify new and more reliable disease-linked biomarkers to stratify BLCA patients into well-defined risk groups to enable better disease management and treatment.

The first member of the nuclear factor of the activated T-cells (NFAT) family was discovered when T lymphocytes were stimulated by antigens to activate gene transcription leading to new cytokine synthesis of e.g. interleukin-2 [6]. Simultaneously, Feske and colleagues found that the activation of immune cells causes a rise in cytosolic calcium ion levels which cause NFAT activation *in vivo*, promoting the subsequent immune response to pathogen infection [7]. Surprisingly, recent studies suggest that NFAT family members can regulate cancer development and metastasis. In melanoma cells, NFAT2 and NFAT4 are activated by B-RAF-V600E via the canonical MAPK signal transduction pathway to promote COX-2 gene expression [8]. Increased COX-2 expression is associated with poor prognosis in cancer [8]. In breast and colon cancer, NFAT5 can promote cancer invasion via the involvement of integrin α_6_β_4_ [9]. In breast cancer, NFAT activity promotes the invasion by stimulating COX-2 expression and prostaglandin synthesis [10]. Although NFAT family linkage to tumour development and progression was initially linked to cancer cell proliferation and migration, the prognostic value of NFAT activity in BLCA patients was unclear.

Bioinformatics has become an increasing and widely used tool for tumor diagnosis, prognosis and prediction in cancer cases. Using a bioinformatics approach, Thakur and colleagues showed that transcriptomic signatures could have a prognostic value in melanoma [11]. In another study, screening a set of specific miRNAs using data deposited in The Cancer Genome Atlas (TCGA) database could be used to diagnose oral cancer; this conclusion was further supported from an analysis of the Gene Expression Omnibus (GEO) database within the National Center for Biotechnology Information (NCBI) [12]. Li and colleagues used clinical datasets in TCGA to assess the CpG island methylator phenotype (CIMP) in colorectal cancer and links to genomic aberrations and immune infiltration [13]. Meanwhile, the prognostic model using 4 genes was built to predict the overall survival (OS) of hepatocellular carcinoma (HCC) patients [13].

In this study, we identified NFAT family expression in pan-cancer models using the TCGA database and genotype tissue expression (GTEx). We then combined LASSO regression and Cox regression analyses to build a predictive model for BLCA patient prognosis. A 5 genes prognostic model was established which included NFAT2, FER1L4, RNF128, EPHB6 and FN1. This model was validated by analysis of different BLCA clinical datasets from multiple databases. This study supports the use of the NFAT family as a prognostic biomarker to help in BLCA stratification, disease management and therapy.

## MATERIALS AND METHODS

### Pan-cancer profiling for NFAT family gene expression

To analyze gene expression profiles for the NFAT family of gene products in different malignant cancer, the GTEx, TCGA, and Oncomine databases were used. Oncomine is an online cancer microarray database (http://www.oncomine.org) [14] with a gene chip-based database and integrated data extraction platform. In this study, Oncomine was selected to compare the gene expression of NFAT family in tumors vs. normal tissues. The selection criteria for this study were “*P*<0.05, threshold: 2-fold change, gene rank: top 10%.” Meanwhile, the Gene Expression Profiling Interactive Analysis (GEPIA) tool was used to analyze the clinical datasets in the GTEx and TCGA databases to compare NFAT gene expression differences in pan-cancer models (http://gepia.cancer-pku.cn) [15].

### Analysis of NFAT genetic alterations and expression profiling

We utilized cBioPortal which is a visualization website integrating data from 126 tumor genome projects (http://www.cbioportal.org) [16]. We specified our query tumor type as “bladder urothelial carcinoma” and gene query names “NFAT1, NFAT2, NFAT3, NFAT4 or NFAT4” were selected on the cBioPortal server. The NFAT mRNA expression levels were analyzed and Kaplan-Meier (KM) survival curves were generated to evaluate NFAT expression on overall survival (OS) and disease-free survival (DFS).

### Kaplan-Meier analysis

To analyze BLCA prognostic values, R software with survival package was used to display OS. The P-value was calculated with values below 0.05 considered statistically significant.

### Analysis of differentially expressed genes (DEGs)

The clinical data were extracted from the TCGA database and divided into high expression and low mRNA expression groups according to NFAT2 median expression. R software with limma, pheatmap, and ggplot2 packages was used to determine differential genes between the two groups. The 20 DEGs with the most significant up-regulation and down-regulation were displayed using volcanic and heat maps. Differential gene screening criteria | log FC | ≥2, *P*<0.05.

### Analysis GO and KEGG pathway

R software with clusterProfiler, DOSE and enrichplot packages was used to perform gene ontology (GO) functional analysis and Kyoto Encyclopedia of Genes and Genomes (KEGG) pathway analysis on the differential genes, and FDR<0.05 was set as a cut-off for significance [17–19].

### Analysis of independent prognostic factor

R software with survival and survminer packages was used to analyze TCGA clinical data. Both univariate analysis and multivariate analysis were used to generate Cox proportional hazard regression models.

### Protein-protein interaction analysis

The top 100 DEGs were imported into the Search Tool for the Retrieval of Interacting Genes (STRING). This online tool facilitates building protein-protein interaction networks (PPI) (https://string-db.org) [20]. Results were also imported into Cytoscape software [21], and protein-protein interaction networks and specific nodes within such networks were further analyzed using the MCODE tool in Cytoscape. The network scoring degree cutoff was 2 and the K-core was 2.

### Construction of risk score models

First, the DEGs identified by limma were subjected to univariate Cox regression analysis. Second, a logistic regression model and the LASSO method for variable selection and shrinkage were applied to narrow the mRNA expression profiles by using R package glmnet 4.0. The penalty regularization parameter k was determined via the cross-validation routine before running the main algorithm with an n-fold value equal to 10. The k value was finalized by using lambda min, which was the value of lambda, giving minimum mean cross-validated error [22]. Then, the data were randomly divided into training and testing sets. Finally, the multivariate Cox regression model was built. Besides, the model based on the training group was validated in the testing group by ROC and nomogram. Nomograms were widely applied to predict cancer patients’ prognoses, mainly because they could reduce the statistical prediction models into a single numerical assessment of the probability of OS that was tailored to the individual patient’s profile. In this study, the combined model based on all independent prognostic factors selected by the multivariable Cox regression analysis was used to construct a nomogram to assess the probability of 1-3-5 years OS for patients with BLCA. Subsequently, the nomogram’s calibration curve was evaluated graphically by plotting the nomogram prediction probabilities against the observed rates. Overlapping with the reference line demonstrated that the model was in perfect agreement.

### Analysis of GEO database

We downloaded data from GSE13507 and GSE48276 in the GEO database in NCBI (http://www.ncbi.nlm.nih.gov/geo) [23]. This included data from 256 samples of bladder cancer tissue and adjacent tissues. GSE13507 is an expression profiling study with 165 primary bladder cancer samples, 23 recurrent non-muscle invasive tumor tissues, 58 normal-looking bladder mucosae surrounding cancer and 10 normal bladder mucosae analyzed using an Illumina human-6 v2.0 expression bead chip platform. R software was used to verify the risk model obtained by analysis of TCGA datasets.

### Analysis of immunohistochemistry expression

The Human Protein Atlas (HPA) database is a large-scale protein research project, the main purpose of which is to map the positions of proteins encoded by expressed genes in human tissues and cells (https://www.proteinatlas.org) [24]. IHC data for potential clinical application was extracted from the HPA database in both normal tissue and bladder urothelial cancer, and results were shown as typical images. Such data validated the potential significance of NFAT2 in bladder cancer prognosis prediction.

### Cell culture

Human bladder cancer cell lines T24, 5637, and J82 were obtained from the Cell Bank of Type Culture Collection (Chinese Academy of Sciences, Shanghai Institute of Cell Biology, Shanghai, P. R. China). Cell lines were maintained in RPMI1640 medium (GIBCO, Gaithersburg, MD, USA) supplemented with 10% fetal bovine serum (GIBCO). Cell lines were incubated at 37° C in an atmosphere of 5% CO_2_ and 95% air.

### Colony formation assay

200 cells per well were plated in 6-well plates. After 14 days of culture in RPMI1640 medium, cells were fixed with 4% (w/v) paraformaldehyde, washed with PBS and stained with 0.1% (w/v) crystal violet.

### Cell proliferation assay

Cell proliferation assays were performed using the Cell Counting Kit-8 (Donjindo, Japan). Cells were plated into 96-well plates in triplicate at approximately 2000 cells per well and subjected to different treatment conditions. The OD value was measured using a microplate reader (Thermo Fisher, Waltham, MA, USA) at a wavelength of 450 nm.

### Statistical analysis

The data were presented as the mean ± standard deviation. Unpaired t-tests were used to compare the difference between two groups. *P*<0.05 was considered to indicate a statistically significant difference between the two groups.

## RESULTS

### NFAT family gene expression and genetic alterations in bladder cancer

**Figure 1:**
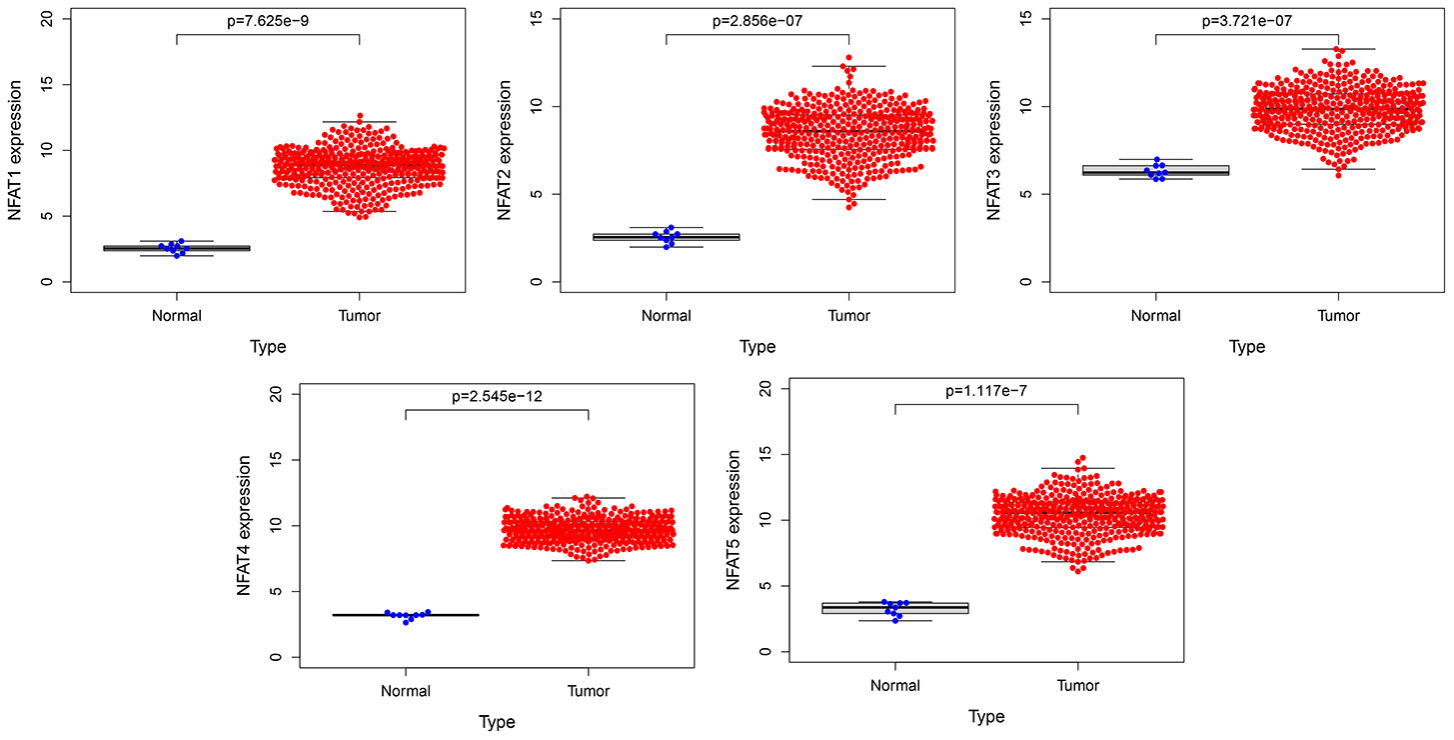
**The expression of NFAT family gene in BLCA.** Blue represents the expression of normal tissues in the GTEx database, and red represents the expression of BLCA patients in the TCGA database.

**Figure 2:**
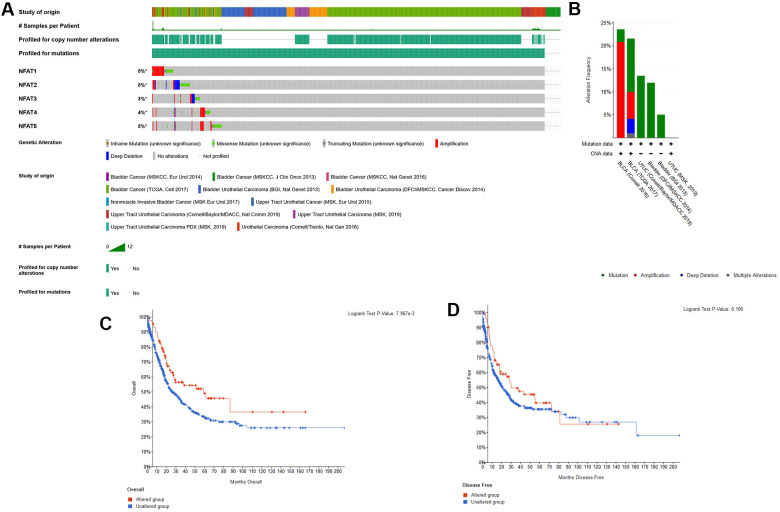
**Genetic alterations of NFAT family genes.** (**A**) Oncoprint visual summary of genetic alterations in NFAT family members. (**B**) Summary of genetic alterations in NFAT family members. (**C**) Kaplan-Meier survival curves for OS in cancer patients with genetic alterations. (**D**) Kaplan-Meier survival curves for DFS in cancer patients with genetic alterations.

The expression of the mRNA expression levels corresponding to NFAT family members in BLCA patients was analyzed using the GTEx and TCGA database. The results showed that the NFAT family gene expression increased in the BLCA (Figure 1). To further determine the genetic alterations linked to such effects we used the cBioPortal database. Amongst the 12 clinical datasets which were analyzed, the frequency of gene alterations, including mutations, amplifications, deep deletions, and multiple alterations, ranged from 0.97% to 20.83%, with mutations and amplifications being the most observed alterations (Figure 2A). The % of genetic alterations within the NFAT family in BLCA varied from 3% to 5% for individual NFAT gene loci (Figure 2B). Meanwhile, clinical survival information was extracted to analyze the prognostic roles of the NFAT family in BLCA patients with or without alterations (Figure 2C). The results showed that the altered group had improved OS, but did not observe any significant correlation between the presence of alterations and DFS (Figure 2D).

### Prognostic value of NFAT family members in BLCA

**Figure 3:**
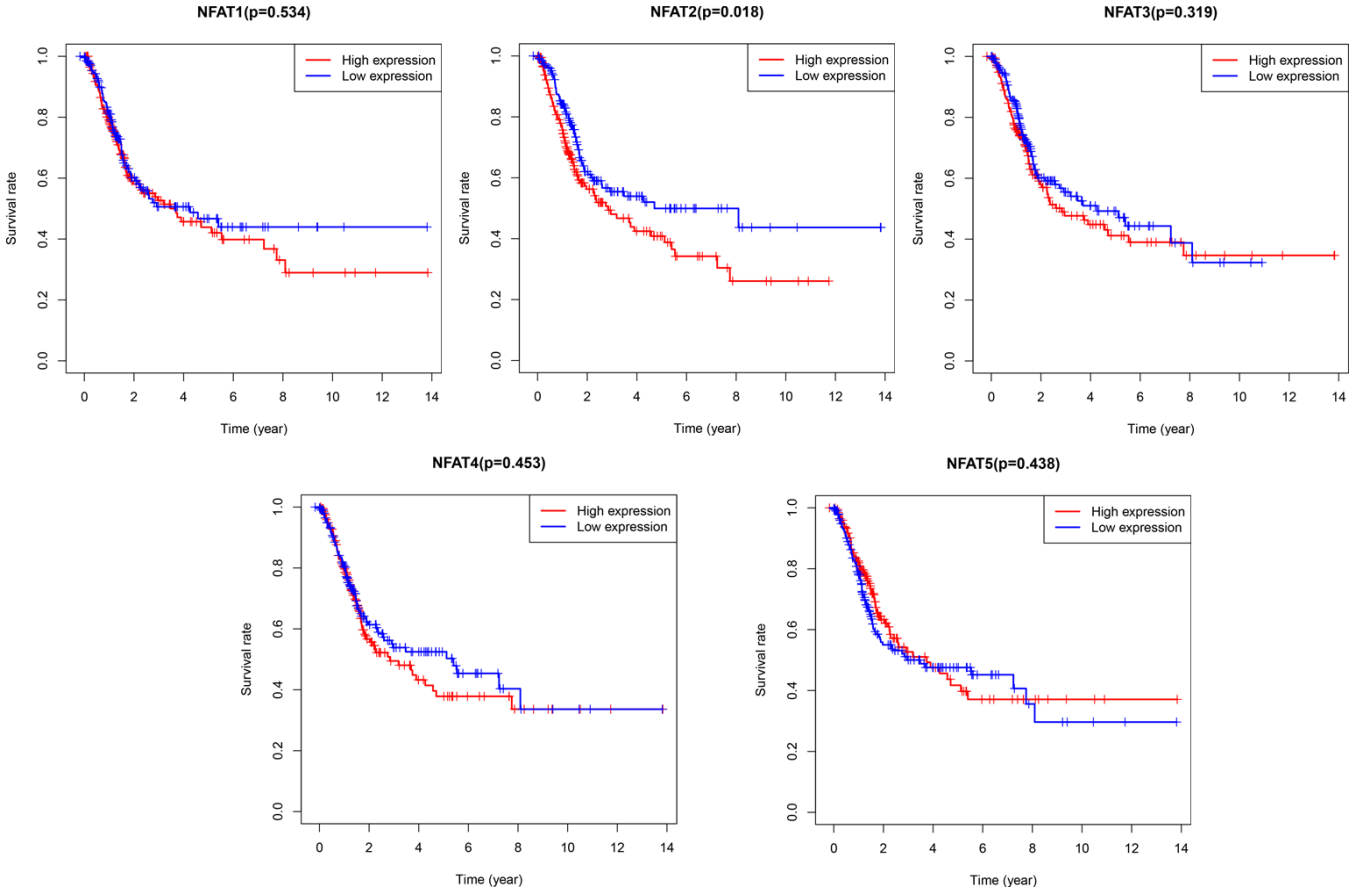
**Prognostic value of NFAT members in BLCA patients.** Kaplan-Meier survival curves for OS of BLCA patients with expression of NFAT1, NFAT2, NFAT3, NFAT4 and NFAT5.

To further explore the prognostic value of each NFAT gene in BLCA, we used R software with a survival package to analyze the clinical BLCA datasets in the TCGA database. The results showed that after grouping BLCA patient overall survival (OS) based on the median value of gene expression, low NFAT2 expression was strongly associated with the better or improved OS, whereas NFAT1, NFAT3, NFAT4, and NFAT5 expression were not related to OS in BLCA patients (Figure 3).

### Expression of NFAT2 in BLCA patients with different clinicopathological features

**Figure 4:**
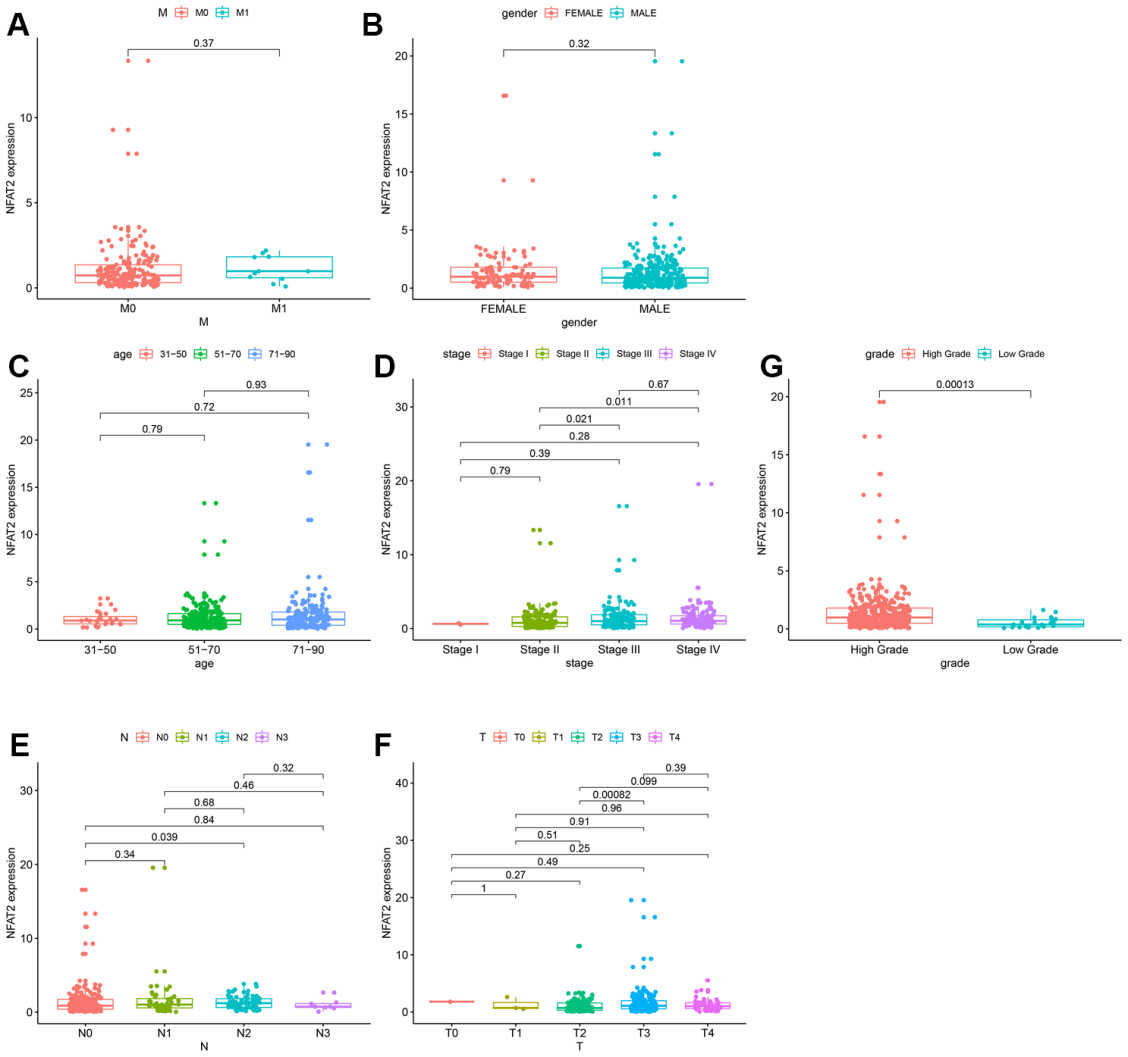
**Expression of NFAT2 in BLCA patients with different clinical and pathological features.** Kaplan-Meier survival curves for OS of BLCA patients and clinical factors. (**A**) M Stage. (**B**) Gender. (**C**) Age. (**D**) Stage. (**E**) N Stage. (**F**) T Stage. (**G**) Grade Stage.

Analysis of the clinical data extracted from TCGA found that the expression of NFAT2 had no relevance to the M stage, stage, gender, and age of the tumor (Figure 4A–4D). For N stage, only N0 and N2 had a significant difference, but there was no continuous significance from N0 to N4. It indicated that NFAT2 and N stage were not closely connected (Figure 4E). The same result also appeared in the T stage (Figure 4F). Surprisingly, in the classification of the BLCA tumor grades, NFAT2 expression in high-grade groups was significantly higher than in low-grade groups (Figure 4G).

### NFAT2 is an independent prognostic risk factor for BLCA

**Figure 5:**
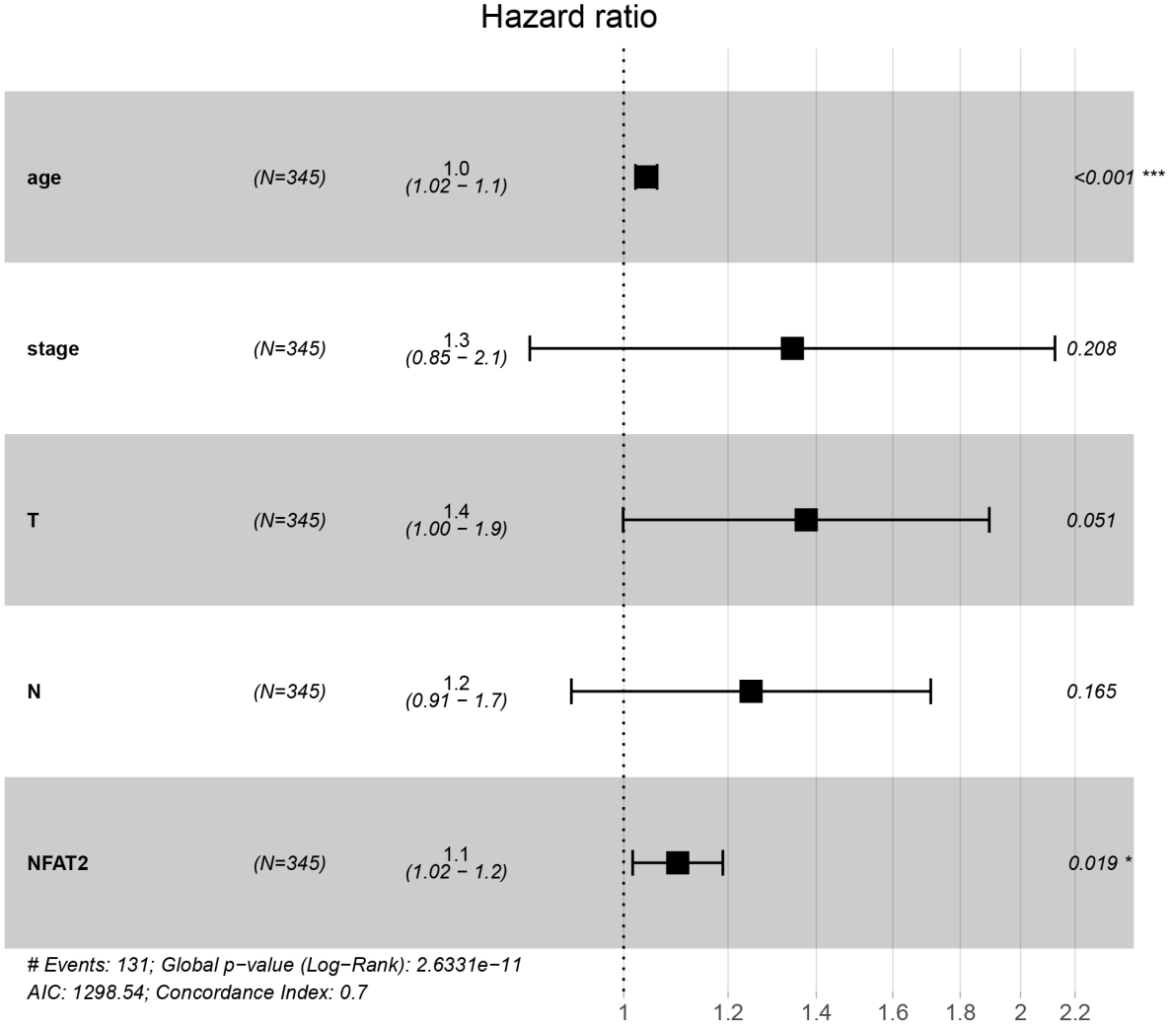
**NFAT2 is an independent prognostic factor of BLCA.** Multivariate Cox proportional hazard model of the expression of NFAT2 and clinical factors.

The information of BLCA patients with incomplete follow-up was removed from subsequent analyses. A Cox regression model for univariate analysis was used: we found that NFAT2 had no significant correlation with BLCA tumor stage and grade (Table 1). Moreover, the hazard ratio (HR) value was 1.113. In the TCGA database, the level characteristics of BLCA were only divided into high-grade and low-grade tumors. However, no deaths were recorded in BLCA patients with low-grade tumors. Surprisingly, multi-factor analysis using the Cox model found that NFAT2 could be used as an independent prognostic risk factor and was not affected by other factors (Figure 5).

**Table 1:** Univariate Cox proportional hazard model of the expression of NFAT2 and clinical factors.

**Univariate Cox analysis**				
	**HR**	**HR.95L**	**HR.95H**	**pvalue**
age	1.041097238	1.02210766	1.06043962	1.80E-05
gender	0.931539367	0.636464096	1.363416409	0.715185647
grade	9617820.06	0	Inf	0.992093304
stage	1.953870349	1.526479865	2.500923482	1.05E-07
T	1.711736629	1.317903959	2.223259342	5.60E-05
N	1.603321291	1.3433964	1.91353733	1.69E-07
M	2.116864599	0.762125873	5.879758041	0.150207498
NFAT2	1.113013537	1.027802607	1.205288958	0.008419016

### Screening for DEGs within BLCA patient groups with differential NFAT expression

**Figure 6:**
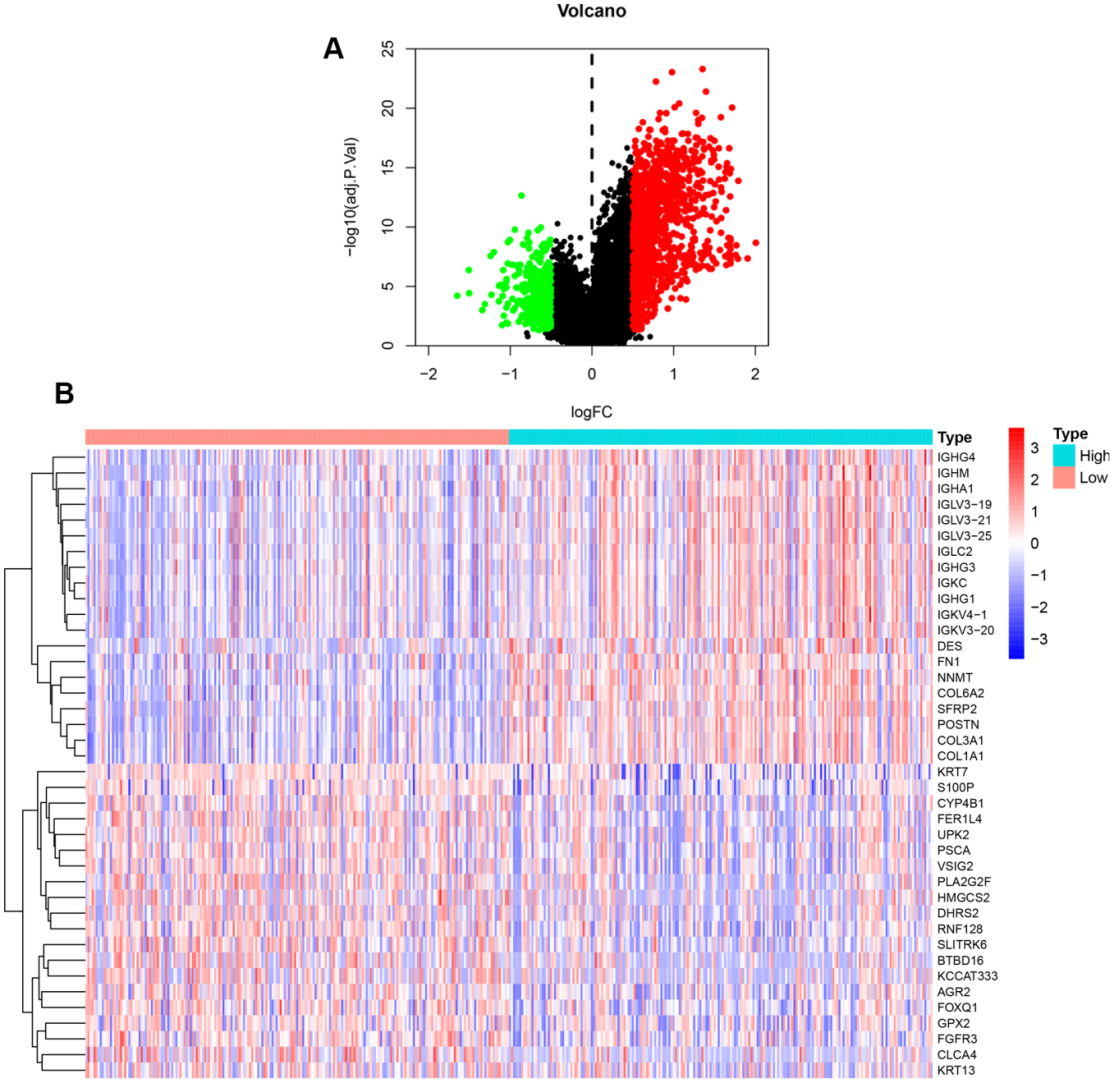
**DEGs between high and low expression of NFAT2 groups.** (**A**) Volcanic map for the DEGs identified by R software with limma package. The abscissa represented log2FC, and the ordinate represented the negative logarithm of the P-value. The red, green, and black nodes represented upregulated mRNA, downregulated mRNA, and non-differentially expressed mRNA. (**B**) Heatmap for the DEGs identified by R software with limma package.

Our previous results demonstrated that NFAT2 expression is linked to OS of BLCA patients. R software with the limma package was applied to screen DEGs of TCGA datasets focused on NFAT2 low vs. high expression groups in BLCA patients. A total of 1447 DEGs were identified with 1153 up-regulated genes and 294 down-regulated genes (Figure 6A). Furthermore, the top 20 up-regulated and down-regulated DEGs were plotted in a heatmap (Figure 6B).

### Biological analysis of DEGs between the high and low expression groups

**Figure 7:**
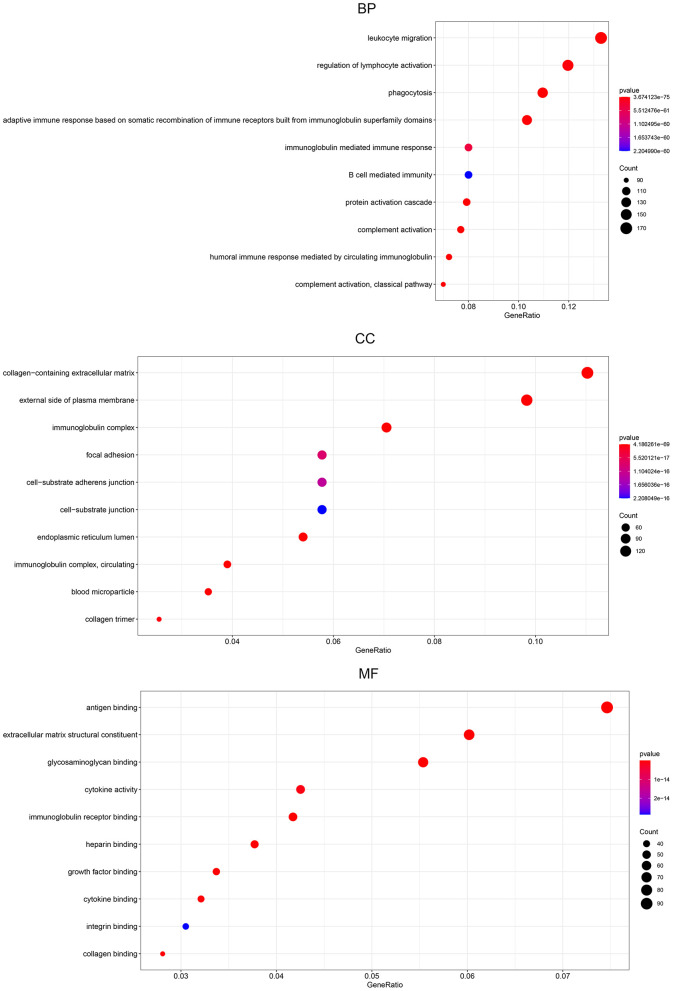
**Gene ontology pathway enrichment analysis of DEGs.** The rich factor demonstrates the degree of enrichment by GO. The node size represents the number of selected genes, and color represents the P-value of the enrichment analysis. CC, cellular component; MF, molecular function; BP, biological process.

To assess DEG functionality, R software with the limma package was used to analyze all DEGs identified in BLCA. Our results showed that 2198 GO terms were enriched. The enrichment items were classified into 3 functional groups: biological process (BP) group (1888 items), cellular component (CC) group (137 items), and molecular function (MF) group (173 items). The top 10 significant biological processes GO terms are shown for each item (Figure 7). Within the BP group, the top 3 significant biological processes were leukocyte migration, regulation of lymphocyte activation, and phagocytosis. In the CC group, highlighted gene products were associated with the collagen-containing extracellular matrix, extracellular side of the plasma membrane, and immunoglobulins. Finally, the most enriched GO terms in the MF group are antigen binding, extracellular matrix structural constituent, and glycosaminoglycan binding.

**Figure 8:**
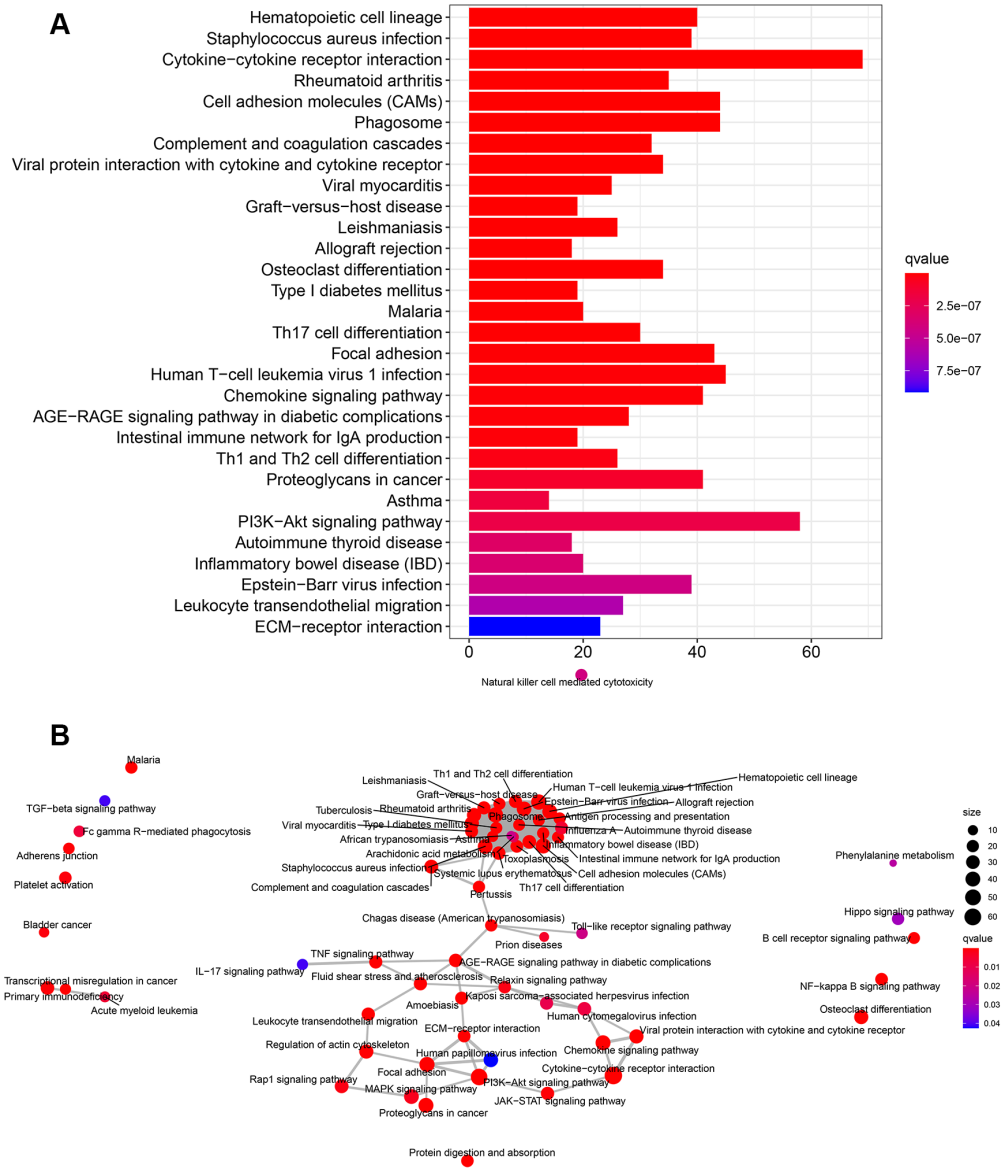
**KEGG pathway enrichment analysis of DEGs.** (**A**) The rich factor demonstrates the degree of enrichment by GO. The Node size represents the number of selected genes, and color represents the P-value of the enrichment analysis. (**B**) Network diagram provides the KEGG pathway interaction in the DEGs.

KEGG pathways were mainly enriched in cytokine-cytokine receptor interaction, human T-cell leukemia virus 1 infection, MAPK signaling pathway, PI3K-Akt signaling pathway, and chemokine signaling pathway (Figure 8A). The pathway-pathway network showed the relationship between the 67 KEGG pathways enriched in all DEGs (Figure 8B).

### Protein-protein interaction (PPI) network analysis

**Figure 9:**
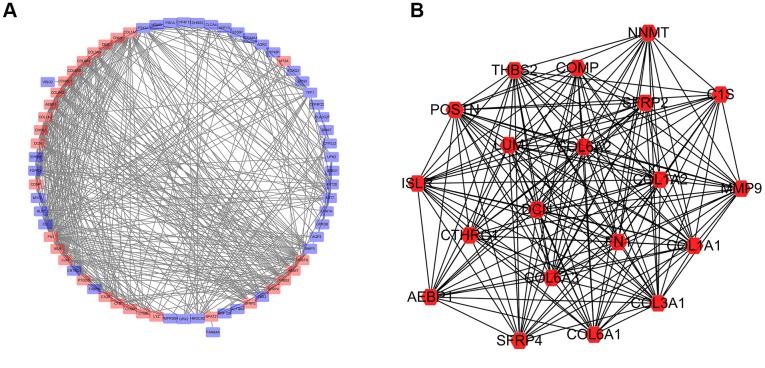
**Network of protein-protein interactions (PPI) analysis.** (**A**) Protein-protein interaction network was constructed for the DEGs using Cytoscape. (**B**) Subnetwork with the highest score using MCODE tool.

Protein-protein interaction (PPI) networks generated *in silico* help us to explore molecular mechanisms associated with specific gene products and biochemical pathways. The interactions among the identified top 100 DEGs were analyzed by using the STRING database. The PPI network of the DEGs consisted of 72 nodes and 533 edges (Figure 9A). Amongst these gene products, C1QA, C1QB, C1QC, COL1A1, and COL1A2 showed the highest combined score in PPI networks, suggesting that NFAT2 plays a key role in the immune system in BLCA. PPI network was imported into Cytoscape. The clusters by the MCODE app showed seven sub-networks, and the sub-network with the highest score is shown in Figure 9B. Other sub-networks were shown in Table 2. These findings suggest that NFAT2 has a significant role cell function which could influence BLCA patient outcomes.

**Table 2:** The 6 sub networks significantly associated with the PPI of DEGs in patients with BLCA.

**Sub Network**				
**Cluster**	**Score**	**Nodes**	**Edges**	**Node IDs**
1	18.211	20	173	MMP9, COL6A3, THBS2, CTHRC1, LUM, COL3A1, COL6A2, COL6A1, SFRP2, COMP, COL1A1, COL1A2, POSTN, NNMT, AEBP1, C1S, ISLR, DCN, SFRP4, FN1
2	5.143	8	18	S100P, PSCA, TFF1, ERBB3, UPK2, AGR2, KRT7, MYCL
3	4	4	6	C1QC, CHI3L1, C1QA, PTGDS
4	3.333	4	5	KRT13, UPK1A, UPK3B, KRT20
5	3	3	3	FOXA1, ID1, FGFR3
6	2.8	6	7	LYZ, KRT19, KLF5, C1QB, CD14, TBX3

### Construction of overall survival risk score model for BLCA

**Figure 10:**
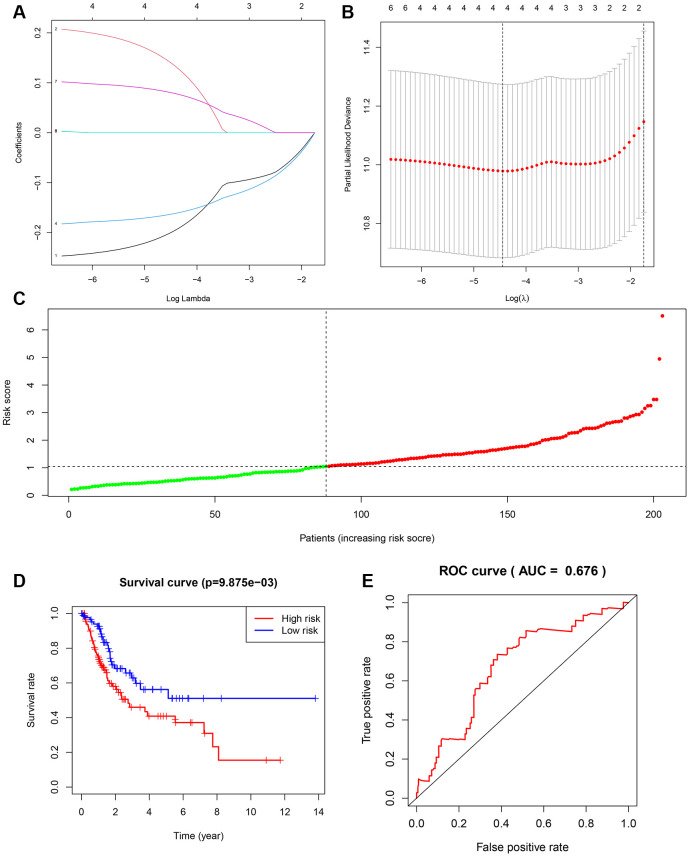
**Construction of overall survival risk score model.** (**A**) LASSO coefficient profiles of the genes associated with the DEGs. (**B**) Partial likelihood deviance was plotted versus log (Lambda). The vertical dotted line indicates the lambda value with the minimum error and the largest lambda value. (**C**) Risk scores of the patients in the high (red) and low (green) risk groups. (**D**) Patients of the validation set from TCGA were divided by risk score into high risk and a low risk groups. OS between two risk groups were analyzed and compared by Kaplan-Meier analysis. Red lines represent the high-risk group samples, and blue lines represent the low-risk group samples. (**E**) ROC curves in the validation set. The abscissa represents sensitivity, and the ordinate represents specificity.

Based on our findings linking NFAT2 expression with BLCA patient overall survival (OS), and NFAT2 risk score model was built to predict BLCA patient survival. Univariate Cox regression was used to assess DEG correlation with NFAT2 in BLCA and we identified 8 gene loci where the P-value was below 0.05. Furthermore, we used LASSO logistic regression combined with 10-fold cross-validation to narrow the mRNA expression profiles (Figure 10A, 10B). As a result, 5 gene loci were identified to build a predictive risk score model.

The samples were randomly divided into training set and testing set, and the training set was used to obtain the model. The predictive model was characterized by the linear combination of the expression of these 5 genes weighted by their relative coefficient in the multivariate Cox regression as follows: risk score = (-0.262991181 * expression of FER1L4) + (0.235961475 * expression of RNF128) + (-0.180646092 * expression of EPHB6) + (0.097234847 * expression of FN1) + (0.084658888 * expression of NFAT2).

The 204 patients of the testing set were used to validate the model in this study. The risk score was calculated, and the median value was used as the cut-off value for the risk score (Figure 10C). The K-M OS curves of the two groups, based on the 5 genes, were significantly different (Figure 10D). The prognostic capacity of the signatures for these 5 genes was assessed by calculating the AUC of a time-dependent ROC curve. The AUC of the prognostic model was 0.673 for the 1-year survival time. These results indicated that the forecast model had high sensitivity and specificity (Figure 10E).

### Evaluation of the risk score model

**Figure 11:**
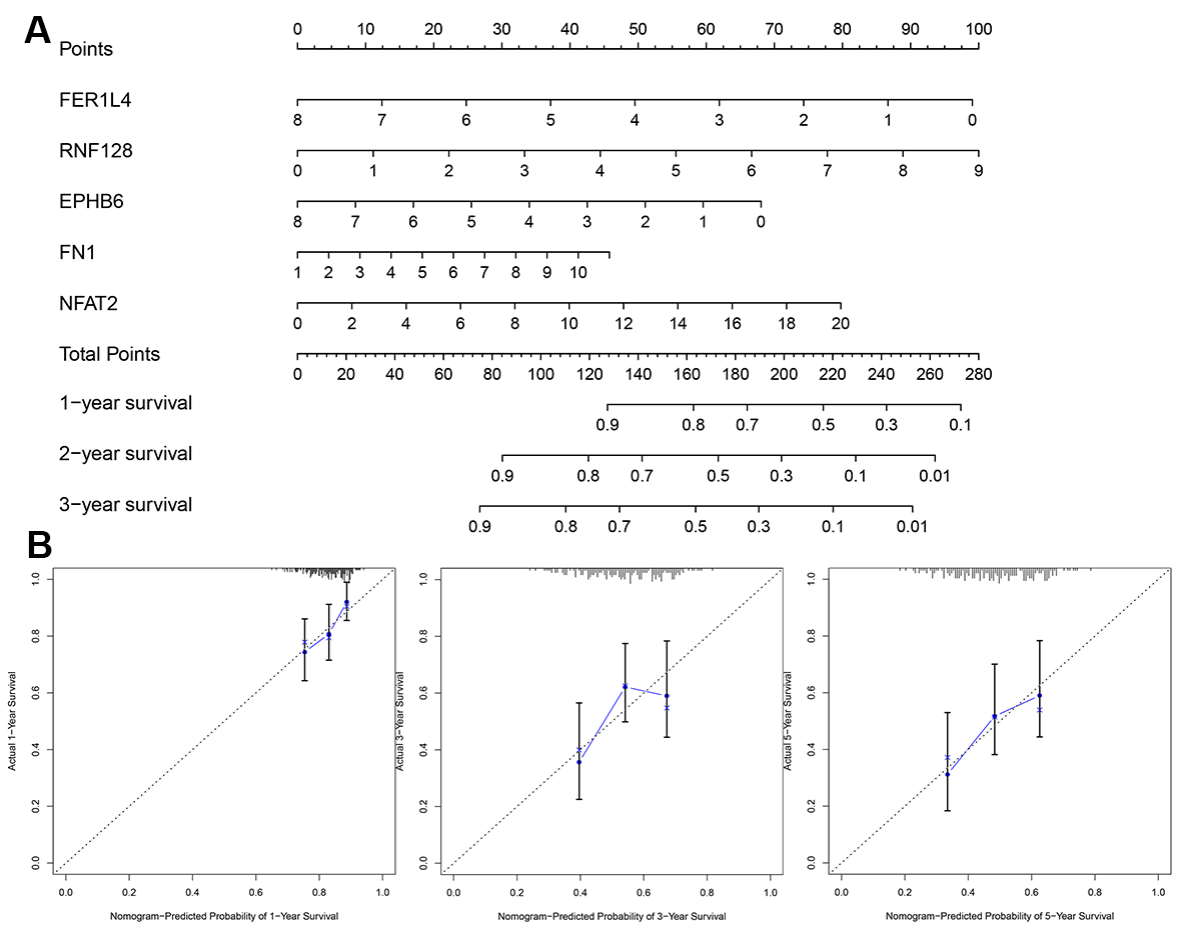
**Evaluation of risk score model.** (**A**) The nomogram is applied by adding up the points identified on the points scale for each variable. (**B**) The calibration curve for predicting 1-3-5 years OS for patients with BLCA. The Y-axis represents actual survival, as measured by K-M analysis, and the X-axis represents the nomogram-predicted survival (*P*<0.05).

To establish a clinically applicable method for predicting the BLCA patients’ survival probability, a nomogram was built to predict the probability of the 1-3-5 years OS in the testing set (Figure 11A). The predictors of the nomogram included 5 factors. The 45° line represented the best prediction. Calibration plots showed that the nomogram performed well (Figure 11B).

### Validation of prognosis risk model

**Figure 12:**
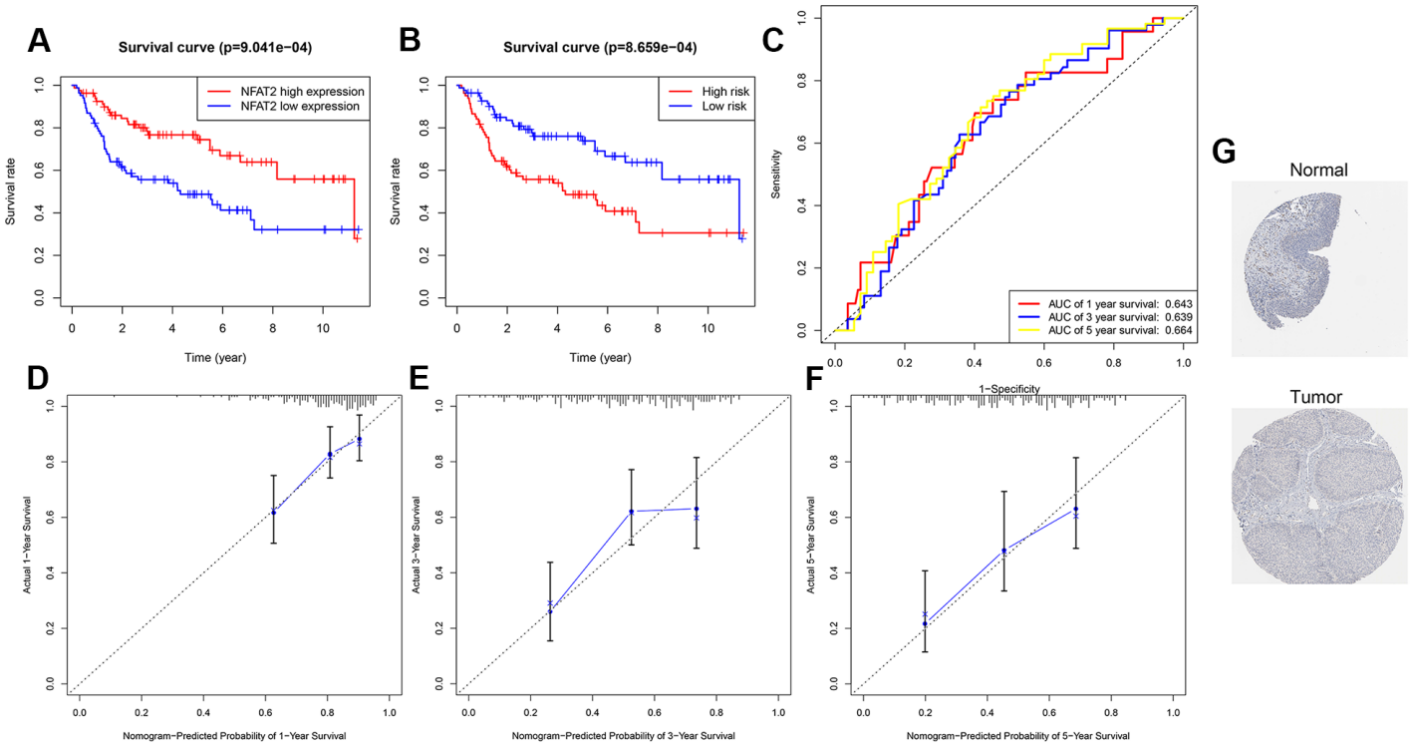
**Validation of prognosis risk model.** (**A**) Kaplan-Meier curves for OS time of patients with expression of NFAT2 in clinical study GSE100926. (**B**) Patients data from GSE100926 were divided by risk score into a high risk and a low risk groups. OS between two risk groups were analyzed and compared by Kaplan-Meier analysis. (**C**) 1-3-5 years ROC curves in GSE100926. The abscissa represents sensitivity, and the ordinate represents specificity. (**D**–**F**) The calibration curve for predicting 1-3-5 years OS for patients with BLCA. The Y-axis represents actual survival, as measured by K-M analysis, and the X-axis represents the nomogram-predicted survival (P<0.05). (**G**) The expression profiles of the NFAT2 in the normal bladder tissue and bladder specimens. Images were taken from the HPA.

To verify the prognostic value of NFAT2 and accuracy of the model for BLCA patients, we further analyzed other BLCA clinical expression datasets. Microarray data from clinical study GSE100926 in the GEO database was extracted and found significant differences in expression between BLCA cancer tissues and adjacent tissues. Representative images of the NFAT2 protein levels were shown the same expression trend in the HPA database. Normal bladder tissue staining of NFAT2 showed median expression in the nucleus (Figure 12G). However, in the cancer tissue samples, the NFAT2 nuclear staining was significantly weaker than in normal tissue. According to the median value of NFAT2 nuclear expression as a classified condition, it was found that the survival condition of the low-expression group of NFAT2 was better than that of the high-expression group, which indicated that the nuclear levels of NFAT2 could affect the survival of BLCA patients (Figure 12A). Meanwhile, the prognostic model was assessed in the GEO data. A total of 165 patients in the GSE100926 data were classified into a low-risk group and a high-risk group using the risk score mode. The result was consistent with the result in TCGA. The OS of the BLCA patients in the GSE100926 data in the high-risk group was significantly lower than that in the low-risk group (*P*<0.05) (Figure 12B). Furthermore, the AUCs of the external verification set were 0.643 at 1 year, 0.639 at 2 years, and 0.664 at 3 years, demonstrating that this risk model can predict the OS of BLCA patients (Figure 12C). The survival of patients was calculated based on the nomogram, and find that the nomogram performed well (Figure 12D, 12E). In order to further verify the accuracy of our model, using the same method with GSE48276 in the GEO database, the results also show that the model has a higher accuracy. The AUCs of GSE48276 was 0.643 at 1 year, 0.619 at 2 years, and 0.622 at 3 years.

### NFAT2 has oncogenic function in BLCA

**Figure 13:**
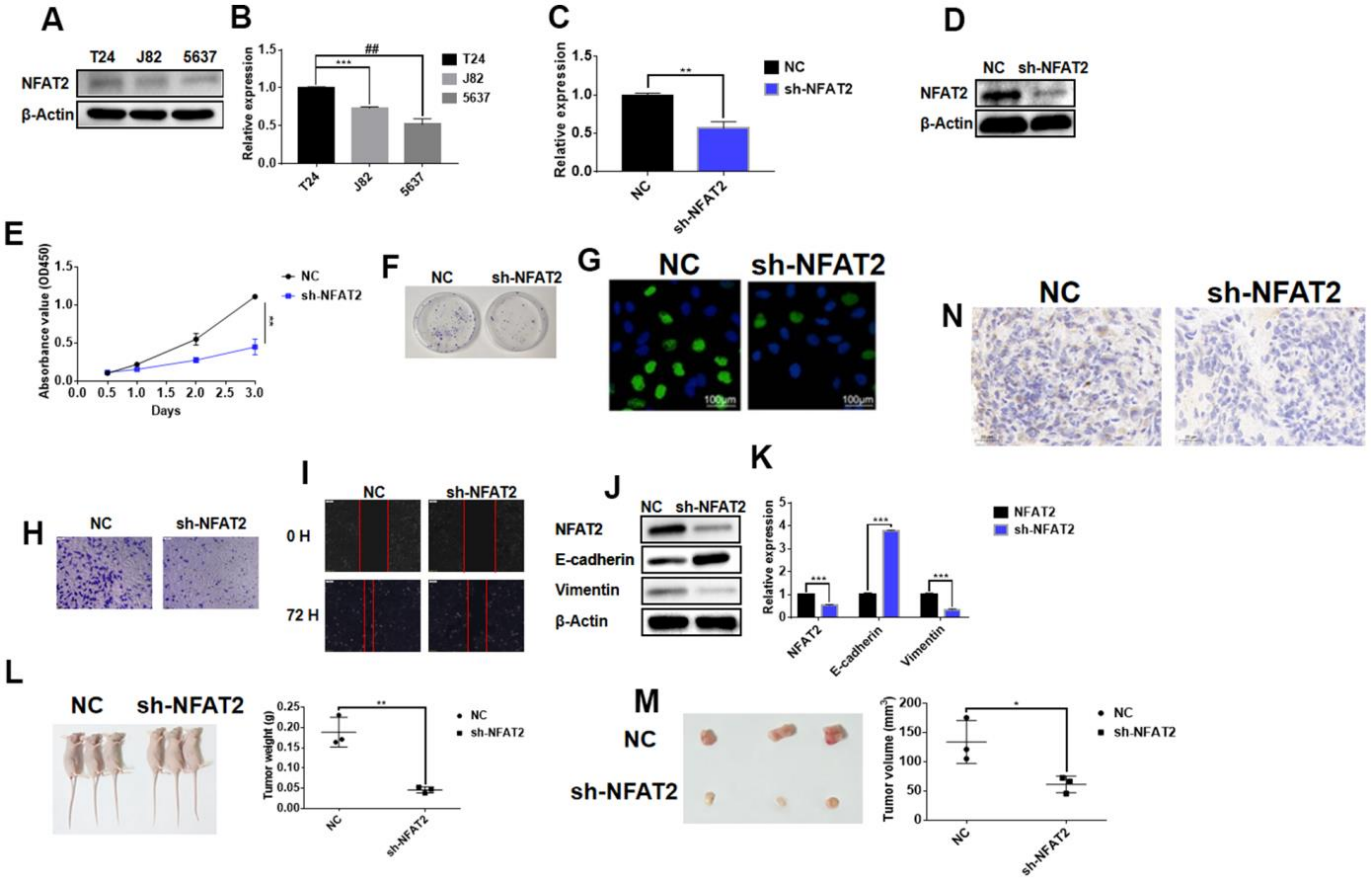
**NFAT2 participates in the regulation of BLCA as an oncogene.** (**A**) The expression of NFAT2 was verified by WB. (**B**) The expression of NFAT2 was verified by RT-PCR. (**C**) The knockdown efficiency was verified by RT-PCR. (**D**) The knockdown efficiency was verified by WB. (**E**) The cell viability was assessed by cell proliferation assay (see Materials and Methods). (**F**) Cell proliferation detected by colony formation assay. (**G**) Cell proliferation was measured by EdU incorporation assay. (**H**) Cell invasion measured by Transwell migration assay. (**I**) Cell migration evaluated by wounded cell monolayer closure assay. (**J**) Western Blot analysis of protein expression of NFAT2 and EMT-linked gene products. (**K**) Quantitative RT-PCR analysis of mRNA for NFAT2 and EMT-related genes. (**L**) Tumor weight from control and NFAT2 knockdown mouse tumor groups. (**M**) Tumor volume from control and NFAT2 knockdown mouse tumor groups. (**N**) Tumor cell proliferation evaluated by Ki-67 immunohistochemical staining. *P<0.05; **P<0.01; ***P<0.001; #*P*<0.05. All data are representative of three independent experiments.

In our previous studies it was found that in BLCA patients, the overall survival of the low expression group of NFAT2 was higher than that of the high expression group. We speculated that NFAT2 may function as an oncogene within the context of BLCA disease. We carried out cellular studies on three different BLCA cell lines, T24, J82, and 5637 (Figure 13). NFAT2 was expressed ~2-fold more in T24 vs. 5637 BLCA cell lines (Figure 13A, 13B). In the 5367 cell line, knockdown of NFAT2 expression using small hairpin RNA (sh-NFAT2) using stable lentiviral transduction caused ~80% reduction in NFAT2 levels (Figure 13C, 13D). NFAT2 knockdown caused ~60% reduction in 5637 cell proliferation (Figure 13E). The colony formation assay showed that knockdown of NFAT2 also caused a significant ~2-fold reduction in formation of the 5637 cell colonies (Figure 13F). Incorporation of EdU nucleotide analog into genomic DNA to assess new DNA synthesis revealed similar findings on 5637 cell proliferation (Figure 13G). Simultaneously, the Transwell cell migration assay showed revealed that NFAT2 knockdown inhibits the migratory and invasive capacity of 5637 cells (Figure 13H). The wounded cell monolayer assay experiment also showed that NFAT2 knockdown caused ~2-fold decrease in cell migration (Figure 13I). Further analysis of control and NFAT2 knockdown 5637 cells using immunoblotting showed a significant increase in E-cadherin and decrease in vimentin levels (Figure 13J). Quantification using qRT-PCR in these 5637 cells revealed ~2-fold decrease in NFAT2 mRNA correlated with ~4-fold increase in E-cadherin and ~4-fold decrease in vimentin mRNA levels (Figure 13K). Such findings link NFAT2 regulation of differential gene expression for E-cadherin and vimentin in BLCA development and progression.

To further assess NFAT2 cancer functionality *in vivo*, the stably transfected 5637 cell line sh-NFAT2 and untransfected 5637 parental control was injected subcutaneously into different groups of BALB/C immunodeficient Nude mice. This was carried out to determine whether NFAT2 expression influences BLCA tumor growth *in vivo.* Comparison of the control and NFAT2 knockdown mouse tumour groups was evaluated: knockdown of NFAT2 caused a significant ~4-fold reduction in tumor weight (Figure 13L) and ~2-fold reduction in tumor volume (Figure 13M). Immunohistochemical staining for the nuclear cell proliferation marker Ki-67 revealed that the NFAT2 knockdown tumor group exhibited reduced staining compared to the control group (Figure 13N). Such findings suggest a strong link between NFAT2 expression and BLCA development and progression *in vivo*.

## DISCUSSION

Bladder cancer (BLCA) is one of the most common cancers worldwide with an increasing incidence of bladder cancer annually. 70% of BLCA patients exhibit tumor metastases after cancer therapy, with a lifetime for follow-up and careful surveillance [25]. At present, BLCA clinical diagnostic methods rely mainly on cystoscopy and urine cytology. Cystoscopy is the gold standard for the BLCA diagnosis and follow-up. However, as cystoscopy is an invasive and expensive clinical technique, it is problematic for BLCA patients. Therefore, there is a need for BLCA biomarkers that have high sensitivity, high specificity, combined with rapid non-invasive diagnostic techniques. Common clinical BLCA biomarkers including urinary fibrinogen/fibrin degradation products (FB/FDP), bladder tumor antigen (BTA), nuclear matrix protein 22 (NMP22), hyaluronic acid (HA), and hyaluronidase (Haase) [26–28] but these still lack accurate disease stratification for BLCA. Therefore, we urgently need to find BLCA biomarkers that are both predictive and can be used early in the disease process. This can help to improve the patient’s postoperative survival time. However, because the period of experimental verification is too long, new biomarkers discovery often takes a long time. The emergence of bioinformatics tools provides us with new ideas for discovering new BLCA biomarkers.

NFAT was first discovered in T -cells as a transcriptional activator of the interleukin 2 [29, 30], and a key regulator of T-cell function in immune responses. Five NFAT family members, including NFAT1, NFAT2, NFAT3, NFAT4, and NFAT5 are often found to play a critical role in regulating the immune system and pro-inflammatory responses. For example, in a mouse model, NFAT activates transcription of the TNFα locus to promote autoimmune diseases such as rheumatoid arthritis, RA [31]. Deletion of the NFAT2 gene locus inhibited mouse thymus development and the expression of anti-apoptotic protein BCL-2 [32]. However, increasing evidence shows a strong link between NFAT family members in tumor initiation, development and progression [8–10]. However, the link between NFAT expression in different cancer patients and link to disease development and progression was unclear. The emergence of cancer bioinformatics provides us with valuable tools for exploring NFAT expression in different cancers and patient outcomes. In our study, by integrating of cancers studies of datasets from GTEx, TCGA, and Oncomine databases, it was found that increased gene expression of NFAT family members correlated with increased incidence of BLCA. By analyzing cancer datasets using cBioPortal, it was found that the alterations within the gene loci encoding NFAT family members could also affect BLCA patient survival. Further analysis of clinical datasets in the TCGA database showed that OS for the NFAT2 low expression group was significantly higher than the NFAT2 high expression group. Multivariate analysis proves that the expression of NFAT2 was not affected by the characteristics of other cases. These findings suggested that NFAT2 might be an independent prognostic risk factor for BLCA diagnosis. R software was used to analyze the function of differential genes between high and low NFAT2 expression groups and construct corresponding interaction networks. It was found that a total of 2198 GO terms, 67 KEGG terms, and 6 sub-networks were enriched. Enrichment into the PI3K-Akt signaling pathway via KEGG was the same as the previous research results. Kim and colleagues have previously shown that nerve growth factor activation of phosphatidylinositol 3-kinase: Akt: glycogen synthase kinase 3β pathway regulates NFAT expression in neurons [33]. Besides, genes in this sub-network are closely linked to the regulation of cell migration. Li and colleagues showed that in ovarian cancer cells, NFATc1 knockdown inhibits cell proliferation and migration [34]. These independent studies further support our hypothesis that NFAT2 promotes BLCA cell migration and proliferation. It is also likely that NFAT2 is involved in other cellular pathways that contribute to cancer development and progression.

An earlier study by Gyorffy and colleagues analyzed clinical datasets from non-small cell lung cancer in the TCGA database using univariate and multivariate Cox regression analysis, Kaplan-Meier analyses, and found that the expression of CDKN2A, OPN, EZH2, ANXA3, ADAM28 and ERCC1 genes significantly correlated with OS [35]. In recent studies, bioinformatics has been widely used to discover biomarkers and the construction of OS and DFS models. Yoshie and colleagues found that in prostate adenocarcinoma (PACA) patients, overexpression of PEG10 is linked to a reduction in PACA patient survival [36]. Another study revealed that the hyaluronic acid family could be used as a BLCA biomarker, whose expression positively correlated with transcriptional regulators such as β-catenin, Twist, and Snail expression levels [37]. In BLCA, patients have a poor prognosis due to the lack of reliable monitoring methods. Therefore, using a reliable prognostic model could help BLCA patients to status better. The use of cancer bioinformatics to build survival models based on multiple gene expression profiles to predict patient survival has been widely accepted and increasingly applied to diverse cancer disease states. Here, we constructed a prognostic risk assessment model based on multiple genes as a basis for clinical treatment. Unlike previous studies, this study not only randomized the original samples, but also identified genes related to prognosis based on an independent prognostic factor NFAT2. Univariate Cox, Lasso and multivariate Cox analyses were conducted to build a risk model to predict risk of BLCA prognosis. FER1L4, RNF128, EPHB6, and FN1 were identified with NFAT2 for prognostic model construction.

For RNF128, the RING finger protein belongs to a subset (RNF subfamily) of the E3 ubiquitin ligase superfamily which facilitates the attachment of ubiquitin and ubiquitin-like proteins to target substrates [38, 39]. More than 200 RNF family members have been identified with diverse properties [40]. Several RNF family members have been implicated in cancer development [41–43]. RNF128 (also known as Grail), as a member of the RNF family, was first discovered as an E3 ubiquitin ligase, which was involved in regulating cellular immune function [44]. However, recent studies suggest that RNF128 plays an essential role in tumor occurrence and development. RNF128 is proposed to ubiquitinate p53 and TBK1 to down-regulate tumor suppressor function and thus promote human leukemia development [45]. In esophageal squamous cell carcinoma, the overexpression of RNF128 promotes signaling through the EGFR/MAPK/MMP-2 pathway to enhance cell invasion and metastasis [46]. In melanoma, down-regulation of RNF128 is proposed to activate Wnt/β-catenin signaling, which promotes higher epithelial-mesenchymal transition (EMT) and cell stemness [47]. RNF128 can also bind other E3 ubiquitin ligases, such as NEDD4, to promote the migration of lung cancer cells [45]. Our mining of the TCGA clinical database found that high RNF128 expression correlated with better BLCA patient prognosis. A model based on the expression of RNF128 was constructed to predict the survival time of BLCA patients.

The ephrin receptor, EphB6, functions as a tumor suppressor in cancer development [48–50]. In breast cancer, low EphB6 expression is linked to enhanced tumor invasiveness; treatment of aggressive breast cancer cell lines with 5’-aza-2’-deoxycytidine elevates EphB6 expression and to reduces tumor cell invasiveness [48]. Hafner and colleagues studied melanomas and found that compared to benign moles, EphB6 mRNA expression decreased in melanoma and metastatic tumors [49]. Another study on colorectal cancer, found that decreased EphB6 expression correlated with decreased OS in cancer patients [50]. However, the link between EphB6 and BLCA disease has not been fully explored. In our studies, the EphB6 low expression group exhibited reduced OS in BLCA disease.

FER1L4 expression could inhibit colon cancer development and progression and could be a prognostic survival indicator in such patients [51]. Furthermore, FER1L4 inhibited the growth and invasion of esophageal squamous cell carcinoma cancer cells [52]. In endometrial cancer, FER1L4 acted as an independent prognostic indicator with the FER1L4 high expression group displayed markedly higher OS compared to the low FER1L4 expression group [53]. Combined with clinical information, we were found that the FER1L4 high expression group of had better OS in BLCA.

Fibronectin 1 (FN1) plays an essential role in cell-matrix and cell adhesion, cell migration, morphogenesis, differentiation, and carcinogenic transformation [54]. In breast and lung cancer, FN1 activated the PI3K/Akt signal transduction pathway by binding to the integrin receptor α5β1 [55, 56]. Han and colleagues found that FN1 stimulated non-small cell lung cancer cell proliferation by activating the mammalian target of rapamycin, mTOR [55]. Similarly, FN1 deletion during colorectal carcinogenesis could inhibit cell proliferation, migration, and invasion [57]. A number of studies have shown that miRNAs that target FN1 could modulate cell proliferation and invasion [58, 59]. Immunohistochemical analysis reporting elevated FN1 expression has been reported in various cancers, including breast, lung, thyroid and esophageal cancer [60–62]. These all indicated that the expression level of FN1 was closely linked to tumor initiation, development and progression.

In terms of the effectiveness and stability assessment of the prognostic model, the AUC of the ROC curve of the internal verification set for the prognostic model for predicting the 1-year survival was 0.673. The AUCs of the external verification set GSE100926 were 0.643 at 1 year, 0.639 at 2 years, and 0.664 at 3 years. Analysis of each dataset shows that the high-risk BLCA group prognosis is worse than that of the low-risk BLCA group. A nomogram was further constructed to predict the OS of BLCA patients, and a calibration chart of the nomogram was drawn. These results showed that this model was a useful predictive model and could be used to predict the survival status of BLCA patients. However, our research also had specific limitations. First, the TCGA database is mainly composed of studies on Caucasians and people of African origin, and further ethnic group studies are required to verify our model. Secondly, our study was based on microarray data analysis. This was needed to extract a single gene to profile NFAT expression in cancer. The mechanism of NFAT2 action requires further studies using cell and animal models.

Overall, our findings indicate the NFAT family expression is closely related to cancer initiation, development and/or progression. In the BLCA disease, NFAT2 can be used as an independent prognostic risk factor in assessing BLCA patient survival. Besides, the 4-factor prognostic model based on NFAT2 was a reliable tool for predicting the OS of BLCA patients. The nomogram provided by this study could be a starting point to better develop personalized treatment plans for BCLA patients.

### Availability of supporting data

The data and files generated during this study are available from the corresponding author upon request.

### Consent for publication

All authors have read this manuscript and approved for submission of the manuscript for publication.
